# Ensuring Quality and Status: Peer Review Practices in Kriterium, A Portal for Quality-Marked Monographs and Edited Volumes in Swedish SSH

**DOI:** 10.3389/frma.2021.740297

**Published:** 2021-10-29

**Authors:** Björn Hammarfelt, Isak Hammar, Helena Francke

**Affiliations:** ^1^ Swedish School of Library and Information Science, University of Borås, Borås, Sweden; ^2^ Joint Faculties of Humanities and Theology, Lund University, Lund, Sweden; ^3^ Gothenburg University Library, University of Gothenburg, Gothenburg, Sweden

**Keywords:** peer review, books, monographs, humanities, social sciences, Sweden, publishing, edited volumes

## Abstract

Although established forms of peer review are often criticized for being slow, secretive, and even unfair, they are repeatedly mentioned by academics as the most important indicator of quality in scholarly publishing. In many countries, the peer review of books is a less codified practice than that of journal articles or conference papers, and the processes and actors involved are far from uniform. In Sweden, the review process of books has seldom been formalized. However, more formal peer review of books has been identified as a response to the increasing importance placed on streamlined peer-reviewed publishing of journal articles in English, which has been described as a direct challenge to more pluralistic publication patterns found particularly in the humanities. In this study, we focus on a novel approach to book review, Kriterium, where an independent portal maintained by academic institutions oversees the reviewing of academic books. The portal administers peer reviews, providing a mark of quality through a process which involves reviewers, an academic coordinator, and an editorial board. The paper studies how this process functions in practice by exploring materials concerning 24 scholarly books reviewed within Kriterium. Our analysis specifically targets tensions identified in the process of reviewing books with a focus on three main themes, namely the intended audience, the edited volume, and the novel role of the academic coordinator. Moreover, we find that the two main aims of the portal–quality enhancement (making research better) and certification (displaying that research is of high quality)–are recurrent in deliberations made in the peer review process. Consequently, we argue that reviewing procedures and criteria of quality are negotiated within a broader discussion where more traditional forms of publishing are challenged by new standards and evaluation practices.

## Introduction

Peer assessment, in more formalized peer review procedures and in less codified contexts such as seminars, is the main evaluation procedure in academia. Peer review comes in many forms and may involve elaborate systems of double-blind review or more loosely organized assessment by editors or colleagues (Horbach and Halffman 2018). The growing emphasis on performance measurement and recurring evaluations of research output have resulted in a greater focus on peer-reviewed publications across all fields. These changes are obvious in the social sciences and humanities (SSH), fields characterized by diverse publication practices and multiple audiences. Indeed, an increasing share of publications marked as “peer-reviewed” has been documented in several countries, including Sweden (Hammarfelt and de Rijcke 2015) and Austria (Gumpenberger et al., 2016). Yet, it remains unclear exactly what peer review denotes in the social sciences and humanities, also in the context of journal publishing (Pölönen, Engels and Guns 2020; Ochsner et al., 2020). While an increase in peer-reviewed output is evident in the social sciences and humanities, publication patterns in terms of genre–journal article, monograph, or book chapter–remain relatively stable. A possible explanation, pointed out by Sivertsen (2016), is that these publication types serve different purposes and engage different methods. Monographs and book chapters account for a large share of all publications and the role and status of the scholarly book remains strong in the social sciences and humanities despite recurring fears regarding the future of academic book publishing (Engels et al., 2018). Moreover, the importance of books, especially single-authored monographs, is great in many fields, which is reflected in peer assessments of candidates for academic positions (Hammarfelt 2017).

The stress on peer review in the assessment of research and the continuing importance of book publishing in many fields have highlighted the need for the development and formalization of peer review of books. For instance, initiatives in Flanders have led to the creation of a “label for peer-reviewed books” (Borghart 2013; Verleysen and Engels 2013), where the status of “peer review” is given to specific titles on a book-by-book basis. The system also offers the status of peer review (“scientific”) to publishers or book series (Giménez-Toledo et al., 2016; Giménez-Toledo et al., 2019), a function that is similar to the Norwegian model (Sivertsen 2018). Similarly, the international initiative DOAB (Directory of Open Access Books) is preparing a “certification service” for peer-reviewed books which will be launched in 2022^1^.

In this paper, we study Kriterium, an initiative that aims to facilitate peer review for books in a Swedish context. Kriterium was launched in the autumn of 2015, and the first six books were published in 2016. It functions as a national portal for administering peer review for academic books by various publishers, including Swedish publishing houses and university publication series. The review process is managed by the Kriterium board, which primarily consists of established researchers, and administered by an academic coordinator who is specially appointed for each manuscript. Inclusion in Kriterium’s series also requires that the accepted book is parallel published open access on Kriterium’s website. As documented by Francke (2017) and Hammar (2017), Kriterium was introduced in response to several concerns regarding the future of publication of academic books in Sweden. A main motivation was to balance what was perceived as an over-emphasis on current evaluation systems in which scholarly books were given little value. Yet, the purpose of launching the platform was also to enhance the quality of scholarly books more generally, to facilitate a more effective dissemination of knowledge, and to encourage open access to books (Hammar 2017). We find Kriterium to be an interesting case for several reasons, including the fact that it acts as an independent platform with the specific role of providing peer review. Moreover, the portal has been developed bottom-up based on a need identified by Swedish scholars in the social sciences and humanities. Finally, Kriterium’s model for peer review has certain unique and innovative features, which we believe are of interest to an international audience.

Based on an analysis of documentation created as part of Kriterium’s peer review process, the aim of the paper is to better understand how the review process and its structure serves to achieve the twin goals of quality enhancement and quality certification of scholarly works. We have paid particular attention to whether there are certain challenges, themes or types of recommendations that are recurring, and that may be specific to the review of scholarly books and to the Kriterium platform. This has led us to focus on how the intended audience is discussed by reviewers; how edited volumes are reviewed; and how the role of the academic coordinator is shaped in relation to authors and volume editors. Following a general introduction to the review of books and to Kriterium, these issues will each be developed in separate sections. Furthermore, with an eye towards the contested role of the scholarly book that prompted the development of Kriterium in the first place, we have looked for instances in the review process where inherent tensions existing in the social sciences and the humanities might become visible.

## Background

### The Peer Review of Books

Although often treated as a uniform process, academic publishing practices differ greatly depending on genre, country, and discipline, albeit the increasingly international academic system leads to more and more standardization. National differences in tradition are apparent not least when it comes to the quality control of books, a topic that has been researched considerably less than that of journal articles (Jubb 2017). In the Anglo-Saxon countries and among publishers working on an international market, it is common for book proposals to be peer reviewed by one or two scholars before a contract is signed, with a second peer review round often taking place once the full manuscript has been submitted (Adema and Rutten 2010; Ferwerda, Pinter and Stern 2017; Jubb 2017). This is a practice introduced by university presses in the 1960s (Pochoda 2012). Furthermore, many publishers will take not only the scholarly quality into account when making their decision, but also the size of the potential audience and commercial value of the book (Jubb, 2017; Verleysen and Engels 2013).

In several European countries, including Sweden, monograph publishing has traditionally not been preceded by blind peer review; rather, scholarly quality is confirmed in other ways (Ferwerda, Pinter and Stern 2017). In some countries, it is common that scholars with a good reputation serve as series editors who make the decision (series editors combined with external peer review is also common in an Anglo-Saxon context). Another model in which the name of the reviewer is disclosed to the audience is the so called “open identify label” used in Central and Eastern Europe. Here, the naming of the reviewer, often a renowned scholar, functions as a quality mark (Kulczycki et al., 2019). In some cases, the academic quality assurance is expected to take place before the manuscript is submitted to the publisher, by colleagues and institutional seminars who read and critique the text as a service to the author(s) and the academic society. For instance, Sweden has a very well-developed internal seminar system in academic departments where texts by doctoral candidates and faculty can be scrutinized before being submitted to various publication outlets.

Countries with small languages, and consequently a small potential audience for academic or professional books, often rely on print subsidies for monograph publishing (Eve 2014; Ferwerda, Pinter and Stern 2017). Subsidies for publishing may be granted by universities or research funders. When applying for production or printing grants, the manuscript may undergo peer review administered by the funder (Björkman 2015), which is one way of confirming quality. The systems for “labels for peer-reviewed books” (Borghart 2013; Verleysen and Engels 2013) mentioned above can be an example of how such diverse quality systems may be standardized to help communicate the level of review both nationally and internationally. In providing a peer review process similar to the Anglo-Saxon tradition for books published by a broad set of often very small publishers and university series, Kriterium fits such a description. The system is concerned only with the scholarly quality of the manuscript. Assessments with regards to audience and potential commercial value are left to the publisher although, as we will show, it is in some cases difficult to discern between scholarly quality and usefulness to and accessibility for an audience.

Although the goal of Kriterium primarily was to raise the status of the monograph by ensuring that they could be peer reviewed, edited volumes have made up an almost equal share of manuscripts processed. Peer review of the edited volume format, however, presents its own challenges, some of which could presumably bring the inherent tension between traditional publishing ideals in the humanities and the quality control associated with peer review to the fore (Ochsner, Hug and Daniel 2013; Kulczycki et al., 2018; Engels et al., 2018). While the writing of book chapters in many disciplines still is a common and appreciated way of publishing results without some of the constraints and demands that come with journal articles (Giménez-Toledo et al., 2016; Sivertsen 2016), there is also uncertainty as to their value in terms of merits and careers (Hammarfelt 2017). Adopting the publishing standards associated with peer review journals could arguably entail abandoning this practice in favor of writing more “rewarding” articles (Hammarfelt and de Rijcke 2015; Giménez-Toledo et al., 2016). However, as pointed out by Engels et al. (2018, 603), “the epistemic culture of most of the SSH makes it unlikely that book publishing would go away.” In light of this, the peer review of edited volumes provided by Kriterium can indeed be seen as a form of hybrid solution; an obvious–but possibly idiosyncratic–attempt to raise the status of book chapters but without losing the advantages of the genre as a whole (Nederman 2005; Edwards 2012).

Compared to journal articles, Giménez-Toledo et al. (2016, 693) note that assessment criteria for books are “fuzzy and unclear.” Peer review of edited volumes seldom, if ever, entail the same level of scrutiny as journal articles (or even monographs) and the practice is far from standardized. As a result, there is great variance between publishing houses (or editors) and individual reviewers. Some peer review processes simply look at the volume as a whole, whereas others might provide more in-depth quality control of individual chapters. There are ostensibly several factors that add to the difficulty in adopting peer review formats for individual journal articles to edited volumes. These include the fact that the latter must deal with multiple authors, often from diverse scholarly backgrounds and traditions brandishing different ideals on, for instance, style, methodology, and theoretical perspectives. Finding reviewers that can cover such a wide range is difficult and the issue of where to place the bar for scholarly rigor might prove challenging. Moreover, whereas articles are commonly written with peer review in mind, edited volumes can often be the result of either workshops and conferences or impromptu discussions within informal networks and between local colleagues, situations that do not necessarily take the publishing end of the process into consideration (Edwards 2012)^2^. This can in turn lead to a situation where a number of chapters (or presentations) must be substantially rewritten or refocused to fit the demands of peer review. To be sure, one might even ask for whom the peer review itself serves a purpose, the editors or the individual authors and, moreover, who the driving force is behind the decision to submit (Williams et al., 2018). Indeed, some authors might even be reluctant to publish their chapter in the edited volume unless it is peer reviewed, while others might be reluctant to provide the extra effort required, leaving editors in a bind. With these challenges in mind, Kriterium presents an opportunity to examine how reviewers handle the genre and whether the idiosyncrasies suggested above raise problems in assessing quality.

### The Development of Kriterium

Today, Kriterium is described as a “portal for review, publication and dissemination of high-quality academic books, in accordance with the principles for open access.”^3^ However, this characterization and purpose only crystallized after years of intense negotiation. In fact, retrospective interviews emphasize that Kriterium grew out of a “conglomerate of ideas”, parallel visions, and multiple objectives that co-existed during the pilot phase of the project (Hammar 2017, 5). At the outset, chief of these was arguably to safeguard the existence of the scholarly monograph in the face of increasing status being awarded the international peer-reviewed article in the humanities. At the same time, due in part to the fact that the pilot phase of Kriterium developed out of broader discussions concerning the need for a national consortium of open access books, an awareness that open access was becoming more important in the academic world was palpable by the originators and influenced the process (Lawrence et al., 2013).

Concerns were raised at this early stage over new publication standards and metrics, including the introduction of “publication points” that might threaten the traditional modes of scholarly publication in the social sciences and humanities. Early ambitions targeted the so called “Norwegian list”, a register of publication outlets whose importance has grown in Sweden over the past decade, and which is used by some academic institutions to evaluate the performance of institutions and faculties (Hammarfelt et al., 2016). When Kriterium was awarded 1 point (on a scale from 0-2) on this list, this fact was used in the marketing of the portal.

Another related motivating factor was the fear that Swedish as an academic language would further diminish if scholars were rewarded for publishing articles in English. This idea coalesced with an ambition to protect the existence of Swedish publishing houses (and the academic series published by universities) aimed at scholarly monographs, and to combat the commercialization of academic publishing (Francke 2017). The Swedish book market for scholarly books is small and diverse, consisting of many small-scale, independent publishers, commercial as well as non-profit, alongside the more traditional publishing houses. The latter tend to have less interest in scholarly literature, although there are exceptions. As noted in a study conducted by the National Library of Sweden, the publishing landscape in Sweden is not characterized by prestigious academic publishing houses or university presses (National Library of Sweden 2019). While university presses have begun to appear in the last few years, their function overall is different from the Anglo-Saxon ones, and they are generally non-profit. For one thing, the market diversity has resulted in a lack of standardized practices as regards peer review, although external readers have been a regular feature. The two publishers that were part of the pilot phase of Kriterium can be described as small-scale (run by one or two people) and commercial, specializing in scholarly books where the bulk of the production costs are covered through subsidies from research funding or publishing grants.

Formally, Kriterium can be described as an intermediary, an infrastructure that provides peer review for authors and publishers who wish their books to have a stamp of quality^4^. In the long run, this service is then expected to elevate the status of the scholarly book vis-à-vis the scholarly article (Hammar 2017). As a national infrastructure, Kriterium is intended to benefit the scholarly community as a whole. The founders consisted of scholars, publication experts, librarians, and independent publishers of academic literature. Initial support came from the universities at Gothenburg, Lund, and Uppsala as well as the Swedish Research Council, The Bank of Sweden Tercentenary Foundation, and The National Library of Sweden, and recently more universities have joined the consortium. The challenges of this project notwithstanding, once this solution was agreed upon, Kriterium began to grow out of its tentative larva phase. Although progress was slow at first, for a number of years Kriterium worked hard to become a recognized “brand” within the scholarly community. That they have to some degree succeeded in this endeavor is evidenced by the increasing number of manuscripts that are submitted as well as the growing number of academic institutions that today support Kriterium.

## Materials and Methods

Peer review procedures and editorial work processes are often secretive, and it may be difficult to access material documenting the process. In this case, Kriterium has generously shared the full documentation of board meetings, including peer review reports, as well as communication between the academic coordinator, the authors, and the editorial board. This is a rich material, consisting of hundreds of items, and for the purpose of this paper we have focused on a selection of the available documents. The focus has been on the process of peer review. Two types of documents, namely reports from reviewers and from academic coordinators, have been singled out for further analysis. It should be noted that many reviewers also make suggestions for changes directly in the manuscript. However, these more editorial types of comments, although important, have not been included in the analysis. Overall, the material consists of the reports available for 24 books: 14 edited volumes and 10 monographs. In a few cases, one of the reports was missing. Seven edited volumes were written in English while the remaining 16 books, including all monographs, were in Swedish. Kriterium mainly targets the social sciences and humanities and the manuscripts analyzed come from a wide range of fields including history, archeology, literature, Romance languages, art history, political science, ethnology, and the history of ideas, and they are often interdisciplinary.

A total of 49 review reports, and 28 recommendations by academic coordinators were studied. For the purpose of pseudonymization, we will refer to the books as monograph a, b, c (e.g., Mon A) and edited volume a, b, c (e.g., Edv A), and as review report (e.g., Mon A: Rev) or coordinator report (e.g., Edv A: AC). A few manuscripts are scholarly editions and collections of letters; genres we considered too unique to include in the study. Although the material we have had access to is not complete, we have deemed that it is substantial enough to provide an insight into the review process at Kriterium. Of the 24 analyzed books, 20 had been published in September 2021, a couple more are accepted for publication, and a few have been rejected or withdrawn.

The secretive, and potentially sensitive, nature of the material makes it important to assert that these documents have been handled and analyzed with confidentiality. Given that the social sciences and humanities in Sweden comprise a comparably small context and that the number of books published is limited, we refrain from including information that may identify a specific book when we present quotes or in other ways report our results. In some cases, the quotes have also been translated into English. The people involved in the process may, of course, be able to identify quotes from documents they have had access to.

The material was analyzed through qualitative content analysis (Elo and Kyngäs 2008). In the preparation phase, review reports and coordinator assessments for each work were identified and organized according to monographs and edited volumes, as initial readings confirmed the suspicion that the character of the reviews were to some extent genre specific. Next, the material was coded through inductive content analysis. This iterative process consisted of careful readings and open coding. The three authors focused their reading and coding on mentions of the audience, edited volumes, and the role of the academic coordinator respectively, as these overarching themes appeared as distinctive in the first readings of the documents. Bringing our analyses together, we then identified important sub-themes and terms that structured our analysis. Finally, in reporting our findings, we use summative analyses of the material and illustrate our findings with quotes from the material.

## Kriterium’s Review Process

Before moving on to the identified themes, we first present and discuss the context in the form of the review process and work routines at Kriterium. This section is based on texts about Kriterium as well as on the analysis of review reports.

**FIGURE 1:**
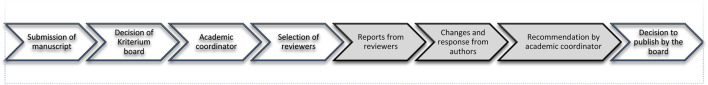
Outline of the review process of Kriterium. The stages marked in grey denote parts of the process that are of particular concern to our analysis (and from which the empirical data have been collected).

The first step in the process of having a book provided with the Kriterium label involves the submission of a book proposal from a publisher or university publication series to the board of Kriterium. The proposal includes a brief description of the book as well as the full manuscript or at least one chapter. If the proposal is deemed to be a suitable contribution (i.e., a scholarly work in book form), the board will appoint an academic coordinator. The book publisher is asked to suggest a suitable coordinator in their proposal. This should be an established researcher within the field of the manuscript, and a person who has not previously collaborated or are colleagues with the author(s). If the book is suggested by a university publication series, the series’ editor will often act as academic coordinator, while the role will be filled by an “ad-hoc” coordinator if the book is to be published by a publishing house. The academic coordinator will suggest two reviewers who will read the full manuscript, be a link between author(s)/editor(s) and reviewers, and make sure that recommendations made are considered when the manuscript is revised by its author(s) (the role of the academic coordinator is further discussed below). Thus, although authors/editors may be involved in suggesting the academic coordinator, it is the coordinator who identifies suitable, independent reviewers, and the review process is either single or double blind. While the ambition in most cases is that authors and reviewers should be unknown to each other (double blind) it remains difficult to uphold the anonymity of authors — and possibly also of reviewers — as the social sciences and humanities community in Sweden is rather small. Still, academic coordinators and reviewers do not need to be — and often are not — Swedish, but obviously it is required that they are literate enough in the language of the manuscript to be able to assess it. Hence, international reviewers are often used for English language manuscripts and for contributions in Swedish it is not uncommon with coordinators or reviewers from Denmark or Norway. After one or two rounds of review, the coordinator will write a recommendation to the board of Kriterium, who then makes the final decision on acceptance. This process and the actors involved are schematically illustrated below (Figure 1)^5^.

Generally, but not always, the review reports are structured according to the instructions given to reviewers. The interface for submitting the reports reflects the criteria used for assessing the quality of the manuscript, consisting of the following headings: “Knowledge claims and themes,” “Structural and linguistic clarity,” “Method-theory-empirical data/sources,” “Scientific context,” “Recommendation and summary of proposed changes,” and “Detailed comments and suggestions.” In addition to these headings, reviewers are given the opportunity to write comments directly to the authors and the publishers, and they are also asked to anonymize their report and disclose any “competing interests.” Despite this structuring of the review process, there is great variance both in terms of length and focus in the reports. The same applies to the style; a few reports show similarities to a published book review, whereas other reports follow the structure envisaged above more closely. These observations correspond well with earlier findings which suggest that instructions for reviewers have little influence on how the actual reports are written (Langfeldt 2001).

## Findings

An overarching question that emerged from our reading of the material was how reviewers and academic coordinators relate to the function of peer review in terms of gatekeeping and/or feedback and with attention to various audiences. In the following, we will focus on three themes that shed light on this broader question through the localized example of Kriterium. The first theme concerns the intended audience and how reviewers navigate possible tensions between intra-academic norms and accessibility for a general audience. The second theme addresses how peer reviewers approach the task of assessing edited volumes; here we find the navigation between the parts (chapters) and the whole volume particularly interesting. The third theme reflects on the role of academic coordinators and reviewers and highlights different interpretations of what these roles entail.

### The Scholarly Book and its Multiple Audiences

The first theme identified in the analysis of reviewer reports concerns questions regarding the intended audience of the scholarly monograph. Of particular importance in this discussion is how to find a balance between a more popular style of text for a general audience and a more scientific, potentially inaccessible style. One reviewer writes: “It is possible that this way of writing scientifically works in a PhD dissertation, but it does not work as well in a book directed to a general public interested in the topic” (Mon D: Rev). In a similar vein, reviewers often comment that authors “give in to the temptation” of offering too much “historical detail” (Mon C: Rev), and it is suggested that meticulous and lengthy accounts may limit the audience to a “local (not clearly identified, but probably academic Swedish) readership.” [Mon A: Rev (parenthesis from original quote)] Evidently, the potential audience is an important parameter in the assessment of monographs. However, targeting an academic audience as well as the general public could possibly come into conflict with other, more intra-disciplinary, ambitions. These tensions are expressed by one reviewer when writing: “The book appears as a work of scholarship where strengthening the argumentation is perhaps more important than to communicate with the general public.” (Mon D: Rev).

Against this background, it comes as no surprise that comments pertaining to style and rigor are prevalent in the reviews. This criterion even has its own heading, “structural and linguistic clarity,” in Kriterium’s instructions for reviewers. Sometimes the text is criticized for being too “heavy” and not very appealing for a general audience (Mon D: Rev), yet there are also instances where the presentation is praised for being “accessible and confident” (Mon K: Rev). At the same time, reviewers put a lot of emphasis on scholarly rigor, careful handling of sources and appropriate referencing. This is of course highly important for a general public because they may be in a less informed position to identify flaws in the text, but an extensive use of references generally characterizes the academic work rather than the popular work of non-fiction. In fact, one of the manuscripts received substantial criticism with regards to referencing, as the reviewers suggest that it was difficult to discern the author’s own contribution in relation to previous literature. Critique was also raised against using translated sources, rather than the original, which was described as “deviating from common scholarly practice” (Mon E: Rev). This suggests that the perspective of the reviews is often primarily a scholarly one, but with an eye towards a broader market.

Generally, in the books studied, reviewers have been recruited from the same field as the author(s), or a closely related one, but several books are of a multidisciplinary nature and there are instances involving reviewers with different disciplinary background. In one case, the reviewer comments on this at the beginning of the report, noting that the comments will be from the reviewer’s disciplinary perspective rather than that of the author (Mon F: Rev). In negotiating disciplinary differences, both before reviewers accept the task and when authors respond to the comments, the role of the academic coordinator is central. An important responsibility of the academic coordinator, much like that of journal or book editors, is thus to be a link between reviewers and authors. This is especially important when handling processes involving several disciplines or schools of thought. Such a task might be challenging with regards to monographs with interdisciplinary content, and even more so for edited volumes that may include chapters from a range of fields, where it is important that each chapter is assessed on its own disciplinary principles. This is expressed by a coordinator when writing their recommendation: “A premise in academic assessment is that each chapter is reviewed within its own paradigm. In the assessment of (author’s) chapter, I believe that the reviewer and the author are part of different paradigms.” (Edv D: AC) When addressing shortcomings in peer review, especially of edited volumes, the responsibility of an editorial function is thus visible in the material.

Consequently, the multiple roles of the scholarly book as simultaneously an intra-disciplinary contribution and disciplinary achievement (merit), a work expected to be read by scholars from other fields, and a means to communicate broadly both within academia and outside it has consequences for how peer review is performed. A balance between scholarly rigor, methodological and theoretical refinement, and an accessible presentation appears to be a key quality sought after in the assessment. Another balance, between parts and whole, is an important theme in the next section which looks at the reviewing of edited volumes.

### Edited Volumes: The Whole Should be Greater than the Sum of its Parts

The edited volume consisting of chapters written by different authors on a specific theme or topic is a popular genre, especially in the humanities. In the social sciences and humanities as a whole, book chapters are the second most common publication type (ranging from 25 to 40% of the total publication output), only preceded by journal articles (Kulczycki et al., 2018). The edited volume has been heralded as important for is function as a “communal and conversational endeavor” (Webster 2020, 35). Yet, the genre is under discussion; the limited reach and impact of book chapters compared to journal articles is highlighted, and the quality of peer review has been questioned (Webster 2020). Such criticism also comes across in the Kriterium reports, for example when a reviewer refers to the general critique which often describes edited volumes as fragmented, lacking a clear focus, or being too narrow in scope (Edv J: Rev). Against this background, we find it interesting to analyze the reviewing of edited volumes in some detail.

The Kriterium review reports of edited volumes, as in the case of monographs, vary in length, focus and adherence to the review topics. As can be expected, a large part of most review reports concerns such aspects as interpretations, previous research, methodology, and theory. However, there are also recurring comments on the format of the edited volume itself that reveal some ambiguities towards quality control and improvement. Often commented on in the reviews is the heterogenic nature of the edited volume. Suggestions for how the manuscripts can better come together as a whole can be found in a majority of the reviews. This lack of coherency has been dubbed the “most common weakness of the edited volume” by Edwards (2012, 64). The basic tension between the whole and its parts is summed up in the following comment in relation to a reviewed manuscript:

Since this is an anthology with more than twenty different contributions, there are variations in the way the treated subjects are approached, from more personal reflexions (sic) to solid and original research. Therefore each article is commented (on) individually in the section *Detailed comments* below, with, in some cases, suggestions of improvement for the benefit of the anthology as a whole (Edv L: Rev).

The quote illustrates some of the challenges of cohesion that reviewers are faced with, even though some reviewers are careful to point out that they believe the plurality of methods and/or perspectives to be beneficial to the manuscript as a whole (e.g., Edv G: Rev). Nevertheless, despite the fact that diversity is arguably built into the genre, many reviewers clearly expect an edited volume to be focused on one topic or theme. One reviewer openly discusses the “challenge” of putting together an edited volume in that individual case studies need to be brought together in a way that makes the volume appear coherent (Edv D: Rev). Interestingly, one reviewer begins with a personal and general observation, claiming that the genre is becoming less prominent with international publishing houses within certain subjects of the humanities and social sciences (see also Edwards 2012; Jubb 2017). According to the reviewer in question, this is mainly due to the fact that reviewers tend to find them “thematically and theoretically sprawling, lacking a common thread and/or too niched”, critical aspects that the reviewer expects the manuscript in question to avoid (Edv J: Rev). The recurring problem of “sprawliness” (Edv K: Rev), “fragmentation” (Edv N: Rev), or “discrepancy” (Edv C: Rev) are manifested in different forms of critique. Here, as in the case of monographs, the audience is taken into consideration. Variations in target audiences and style between chapters are factors that can give a heterogeneous impression. By way of example, one reviewer remarks that some chapters in the volume are written as general introductions while others are “clearly written for an expert audience” (Edv I: Rev).

Other aspects of this theme deal with differences in quality or scientific rigor. One reviewer even suggests that some of the contributors to a volume under review might be looking for a “shortcut” to publishing even though the quality is not up to scientific standards (Edv A: Rev) and adds:

This book aims at collecting the authors under one common aim and that must, in my opinion, be made clearer in each contribution. As it stands, it is transparent that some of the authors of the individual chapters have a different purpose with their contributions than the one for which the editors have brought them together.

How, then, can lack of cohesion be remedied and coherence be achieved? A common way of correcting the sense of incoherence is to suggest revisions of the introductory chapter, described by one of the academic coordinators as “a key chapter to legitimize the volume as a unified, coherent publication” (Edv D: AC. Illustrative examples include a remark that the introduction “needs to be rewritten so that it lays out the point of the book more clearly” (Edv I: Rev) or that “the theme of the book needs to be reconsidered” so that the “common aim of the chapters” becomes clear (Edv A: Rev). One reviewer elaborates on the theme:

This is my greatest reservation about the manuscript. It it (sic) possible that the material might be more suited to two theme issues of a journal. While impressive efforts have been made to frame the material as cohesive in the title and in the Introduction (Ch. 1), Parts I and II do quite different things (Edv G: Rev).

Other suggestions include practical changes to the overall structure, including moving chapters around or changing titles (Edv L: Rev; Edv G: Rev; Edv A: Rev). Individually, authors are at times also encouraged to reference the common topic or theme more clearly or, as in one case, to “align the methodology of this chapter with the stated aim of the book as a whole” (Edv G: Rev). More drastically, one reviewer suggest that three chapters get merged into one in order to improve “structural clarity” (Edv L: Rev). Many reviewers suggest formalizing language, concepts, and tone as well as cross-referencing as ways of achieving a more unified product.

A more radical approach to rectifying lack of cohesion is to suggest the exclusion of certain chapters, due either to lack of quality or thematic connection. One might argue that rejection is indeed a fundamental part of journal peer review but can of course present problems for the editors of an edited volume that is the result of a conference or an initiative among local colleagues. This is put in no uncertain terms by one reviewer via the maxim that a chain is never stronger than its weakest link (Edv M: Rev). In a majority of the fourteen volumes discussed here, at least one of the two reviewers suggests that an individual chapter does not fit the volume’s scope, with several reviews signaling that chapters should be dropped prior to publication. In one particularly critical review, the three initial chapters are said not to “meet the standards” (Edv K: Rev). A couple of reviewers are even so adamant that they cannot recommend publishing unless the chapter is dropped (Edv M: Rev; Edv G: Rev; Edv K: Rev). Others recommend that the author in question should consider alternate publishing outlets (Edv J: Rev; Edv M: Rev). Remarkably, in one review process, however, the two anonymous reviewers suggest different chapters to be excluded (Edv G: Rev). Still, in the cases analyzed here, no volume seems to have actually eliminated a controversial chapter, instead opting for major revisions to the chapter in question^6^.

As shown, the reports reveal points of ambivalence in how to accurately review and revise edited volumes. In some instances, these tensions are discussed more directly in the reviews. One very positive reviewer still claims to “sense some ambivalence in how the project has been put together” (Edv G: Rev; cf. Edv F: Rev) and another proposes that the geographical and institutional background of the contributors needs to be described and motivated “in the introduction in order to avoid the suspicion that the volume adds nothing more than being a (local take on) an existing theoretical framework” (Edv J: Rev). A third example is one reviewer who maintains that although not all of the information provided by the book is “totally new”, this could “indeed hardly be the case in this kind of publication” (Edv L: Rev). Interestingly, one reviewer wants the edited volume to “dare move away from the traditional article” in order to become more appealing for a general audience (Edv A: Rev). Clearly the edited volume poses a challenge for reviewers. These difficulties may be partly attributed to reviewers addressing their comments both to individual authors of chapters and to the editor(s) of the volume. The editor(s) often acts as a link that mediates reviewer comments to the authors, thus having considerable influence on how feedback is received and communicated. In the review process developed by Kriterium we also find another intermediary who oversees the process and the communication between authors, editors, and reviewers. The role of the academic coordinator is the main theme in the next section.

### The Role of Academic Coordinators and Reviewers

The academic coordinators have a somewhat unique role at Kriterium. Although they can be said to perform an editorial role, they generally only do so once and in relation to a manuscript within their field of expertise. They are usually suggested by the publisher (presumably in consultation with the author or volume editors) at the time of submitting a proposal to Kriterium. The Kriterium editorial board approves the choice after ensuring that there is no tight personal or professional link between the actors involved. An exception may occur when the publisher is a university publication series, in which case the series editor, who may be a colleague of the author’s, often takes on the role of academic coordinator^7^.

The work of the academic coordinator is largely similar to that of a journal editor or publisher editor, in that they secure reviewers for the manuscript and ensure that the reviews are received. Moreover, the academic coordinator provides an independent assessment of the reviews based on their own reading of the work in order to advice the authors/volume editors on how to address any necessary revisions of the manuscript. There are several examples of academic coordinators expressing that they take this advisory role seriously, for instance:

The reviewers state fairly different opinions on the manuscript. Both reviewers recommend that it be published, but with minor revisions. In this review, I go through the two review reports and provide a recommendation on revisions the editors should or can consider. Thus, I leave a door open if the editors have very diverging opinions (Edv M: AC).

In the context of academic book publishing, a shift has been identified from the role of the editor as gatekeeper to a more proactive role as commissioning editor, as the editor is also tasked with analyzing business opportunities and recruiting authors to write books that address those opportunities (Dodds 2015; Thompson 2005). Contrary to this development, the academic coordinator, and indeed Kriterium, serve only to support and assure the quality of the manuscript, thus fulfilling a gatekeeper function. The academic coordinators work with the reviewers and the authors/volume editors and in the end make a recommendation to the Kriterium editorial board for the manuscript to be accepted or rejected in the Kriterium series based on an assessment of the manuscript’s scholarly quality^8^. In this way, the role does not involve the final publishing decision, nor considerations about the manuscript’s commercial value although, as discussed above, its suitability to an academic audience is a criterion that is often considered in the academic coordinator reports. Rather, the acceptance decision lies with the Kriterium editorial board, who assess whether the review process has been satisfactorily conducted by peers (the academic coordinator and the reviewers) or not. In this sense, the role of the editorial board is similar to the GPRC system in the Flanders, where a board assess the review process of a publisher, although without being involved in more or less all stages of the process, as is the Kriterium editorial board (Borghart 2013; Francke 2017).

The academic coordinators display varying understandings of their role and these interpretations in turn affect how they interact with the authors or volume editors. The relationship with authors and editors is formally similar in that the academic coordinator generally does not (and should not) know the book contributors (although they are not blind to each other). The interaction is generally initiated after the reviewer comments have been received and the academic coordinator communicates the reviews to the authors or volume editors, along with their own interpretation. A text on the Kriterium website describes the relationship between academic coordinator and author as: “When reviews are completed, the author implements changes to the manuscript in consultation with the academic coordinator.”^9^

The reports to the Kriterium editorial board indicate that some academic coordinators take quite an active role in the work that follows, not least in the case of edited volumes. This is an interpretation of the role we term *the collaborator*, illustrated by an academic coordinator’s report which describes how “contacts have been frequent between the editors and the academic coordinator” (Edv C: AC) to formulate instructions to the chapter authors and ways of communicating revisions. In some cases, the academic coordinator even proposes solutions to the problems identified by the reviewers:

This can also (perhaps in combination) be achieved by more work on the introduction and a division of the various chapters — perhaps with short intros to each part. I couldn’t help but test it, and think about how one could do it, I don’t know if I go too far in my role as academic coordinator but I choose in this letter to make a suggestion (mostly as an example of how one could do — not an example of what one should do) (Edv J: AC).

In another report, the academic coordinator indicates having discussions and sharing concerns with the volume editors with regards to some of the book chapters: “Both I and the editors thought that there were problems with the citation technique” (Edv L: AC). There are occasionally similar descriptions of ongoing contact and discussion between the academic coordinator and the author of a monograph.

In other cases, the academic coordinator seems to take on the role more in terms of a *project manager* than as a constructive partnership. This is the case, for example, when the academic coordinator asks the editors to propose a “strategy and time plan for the revision of the manuscript” (Edv L: AC; cf. Edv G: AC). The line between the collaborator and project manager roles is sometimes blurred, as seen in the following quote where the process of “re-writing” the book manuscript is described: “We decided to have a Zoom conference … to discuss the introductory chapter. And as academic coordinator I must applaud the substantial re-writing ...” (Edv J: AC). Notably, the coordinator in this case see themselves as part of a “we” discussing the revisions, yet at the same time the role is also that of someone making an assessment of the process (applauding).

When the academic coordinators interpret their role as *an examiner,* they are more inclined to provide direct requirements for what they expect to be revised in order for the work to achieve sufficient quality for Kriterium. For instance, one academic coordinator report supplements the reviews with very clear instructions: “This added chapter is a MUST on my part, no matter how well-written R2 considers your current final remarks to be.” (Edv K: AC).

The academic coordinators taking on the examiner role often provide constructive suggestions to the authors or book editors of how the work may be improved, but do so as a requirement rather than by inviting dialogue. A more extensive example is offered below:

Make sure the authors limit their references to all kinds of likely and unlikely idols but please encourage them to thoroughly read the theoretical sections (chapters) in (xx’s) dissertation. Ideally, these would impact the analytical approaches in separate chapters as well as in your discussions (Edv K: AC).

Such requirements as exemplified above occur both at the stage when the academic coordinator’s comments are sent to the authors or volume editors and when academic coordinators report to the Kriterium editorial board on their evaluation of the revisions made. In the latter case, however, the required changes are generally described to be “things that can quickly be remedied” (Mon G: AC). Even so, there are also examples where a “re-write and re-submit” procedure is recommended based on the reviews.

It is reasonable to assume that for some of the reviewers and academic coordinators, the role as reviewer of a book pre-publication is a new experience. Unlike the constructively critical role of a scholarly seminar participant or dissertation supervisor, the reviewer and coordinator partly take on the role of gatekeeper, in that they advise the Kriterium board on whether or not to accept the manuscript. Similarly, there are several examples of reviewers who with little hesitation suggest that a manuscript must be revised (Mon J: Rev) or even rejected (Mon E: Rev), and an important part of the reviewer task is to conclude if a manuscript is acceptable, in need of required revisions, or if it should be rejected. Yet, another role, that of a discussant, or critical friend, also comes across in the reports. In a couple of reports, the reviewers actually discuss their role and the function of peer review in relation to how their recommendations should be viewed:

My intention is that the author should be provided an opportunity to reflect on these suggestions in order to facilitate certain changes. However, without in any way advising against publication or require a new review round. The discussion, even based on the current manuscript, may of course also contribute to the process of increased knowledge and understanding of the questions raised by the study (Mon E: Rev).

Similar views are expressed by an academic coordinator for another monograph:

At the same time the author cannot be criticized for making certain choices; it would be unreasonable to ask for a different book (based on discourse analysis, rhetorical analysis or critique of ideology). The peer review should be read as suggestions for revision, and not as a rejection of the project (Mon D: AC).

In our view, these statements can be related to the integrity held by the “author” in parts of the social sciences and humanities. Indeed, it could be argued that the scholarly author — especially in fields were monographs play a central role — is seen as an individual and unique subject (much like a fiction author). Hence, suggestions for major revisions may threaten the autonomy of the researcher. Reviewers as well as academic coordinators may therefore be cautious when formulating their recommendations.

As the descriptions above have shown, although the primary function of the review process is that of gatekeeping, of ensuring high academic quality, it is also in many cases viewed as a quality-enhancing and supporting process. Several of the academic coordinators express great appreciation at how seriously the authors and book editors have taken the task of revising and how thoroughly they have worked with their texts based on the reviews (see also Hammar 2017). In the reports to the Kriterium editorial board it is pointed out that the process has served to greatly improve the scholarly quality of the book, in addition to establishing that the work is of high scholarly quality. The balancing of these two aims of peer review — *enhancing* and *certifying* quality — is a recurrent theme throughout the material.

## Discussion and Conclusion

The focus of this paper has been on the review process of Kriterium and how it serves to achieve the twin goals of quality enhancement and quality certification of scholarly works. With an eye towards the contested role of the scholarly book that prompted the development of Kriterium in the first place, we have looked for instances in the review process where inherent tensions existing in publishing social sciences and humanities research might become visible, thus contributing to the knowledge about peer review in these disciplines. The first tension arises in relation to the intended audience of especially monographs in the humanities, which are expected to address both a scholarly and a general audience. Hence, in adhering to these expectations a work should both have scholarly rigor (to be given the criteria of “scholarly”) and, at the same time, be of interest to a broader audience. Many of the comments made by reviewers and coordinators, especially on Swedish-language monographs, reflect on this tension, where finding the balance between scholarly achievements in terms of theoretical and methodological proficiencies and an attractive and accessible presentation is of great importance. While we find that scholarliness (including disciplinary jargon) and accessibility may be positioned in opposition to each other, it is also evident that many reviewers and academic coordinators do not see these as mutually exclusive. Indeed, a high-quality monograph in many humanities fields displays high scholarly quality in a style that can also attract non-expert readers.

Contrary to monographs, edited volumes primarily target a scholarly audience and they are more often written for an international audience. In our analysis we find that a particular concern raised by reviewers is how the parts (chapters) relate to the volume as a whole. Typically, discussions involve matters such as how the volume holds together (do all chapters fit), and whether or not all chapters have the quality required to be included in the volume and eventually marked with the Kriterium quality stamp. Interestingly, reviewers are concerned both with individual chapters and the volume as a whole. This marks a difference from many international publishers, where chapters often are reviewed in isolation. By using the same reviewers for the whole volume, Kriterium puts emphasis on the coherence of the book. While such an approach could possibly result in more integrated and coherent edited volumes, it may also give rise to specific tensions in the review process. For example, there are several examples of reviewers suggesting that specific chapters are thoroughly revised, or even dropped from the edited volume completely. However, from what we have observed, chapters are rarely omitted, although they may be revised. This could be viewed as a confirmation of the notion that edited volumes allow for contributions of varying quality and relevance. Such an interpretation would be in line with Webster (2020), who in defending the genre of edited volumes highlights its communal and conversational function. In practice then, the edited volume sometimes offers challenges to the peer review process, as omitting chapters based on low quality and/or relevance would impact the collective character of the genre. Notably, discussions in the material regarding edited volumes are, sometimes explicitly, related to a debate about the role of edited volumes more generally. There are examples of reviewers suggesting that some contributions might have been better suited for another format (for example a special issue for a journal). In all, we find that the reviewing of edited volumes is characterized by negotiations regarding the wider purpose and legitimation of the genre as such.

The academic coordinators at Kriterium play an important role in mediating between authors/volume editors, reviewers, and the Kriterium editorial board. These coordinators perform functions that are similar to an editor of a journal in that they select reviewers and facilitate the review process. Yet, the analysis makes evident that the coordinators sometimes interpret their role in partly different ways from what is generally expected from a journal editor, or even book editor. The coordinator reports illustrate that the academic coordinators interpret their task as being that of a *collaborator*, who will work actively to provide suggestions for improvements, thus contributing to enhancing the quality, as a *project manager*, who tries to facilitate the process of finishing the work, and as that of an *examiner*, who takes on the role of quality gatekeeper. These are all roles that could characterize the book editor, but with the important difference that the academic coordinator performs the task once, and with a focus on quality assurance only, without considerations for the other aspects of book publishing. Notable among both reviewers and academic coordinators is the respect shown for the choices made by authors, which we suggest may be a consequence of the relative status, autonomy, and independence of the author in many humanities and social sciences fields.

In a broader context, Kriterium projects questions about academic publishing patterns. Whereas the social sciences in Sweden have more wholeheartedly embraced the international tradition of peer-reviewed journals, impact factors, and “publication points”, the humanities in Sweden, as in many other countries, are still characterized by more pluralistic patterns of publishing (Lawrence et al., 2013). To be sure, there is great variety between different disciplines (and even universities) within the humanities, and some subjects, such as philosophy, are more adapted to international practices. Nevertheless, many scholars in the humanities have remained hesitant to developments that promote publishing in international journals, for instance arguing publicly that a tradition of academic book publishing for general Swedish audiences is well worth protecting (Östling et al., 2016; Heuman et al., 2020). It is still too early to judge if Kriterium has been successful in safe-guarding pluralistic patterns of publishing. The service has rather quickly established itself as a central actor in the publishing landscape of the social sciences and humanities in Sweden with many submissions and strong support from funders, universities, and libraries. Yet, while the initiative at the outset was, at least in part, focused on the peer review of monographs written in Swedish, a considerable part of the reviewed material now consists of edited volumes in English. Hence, the Kriterium platform offers a unique service in the form of peer-reviewing Swedish-language monographs, yet in terms of edited volumes in English authors have several options, including commercial publishers such as Palgrave or Routledge. In contrast to these, however, Kriterium requires mandatory open access, as well as an assessment of manuscripts which does not involve estimates of sales and profit (which might be the case with the books’ publishers). With gaining popularity, a challenge for Kriterium in the future may be how to successfully define its purpose in relation to other actors in the publishing landscape.

The peer review of scholarly books, both monographs and edited volumes, is still a novel practice within Swedish social sciences and humanities. In light of its origins, Kriterium, interpreted as a response to shifts in academic publishing and their perceived consequences, in many ways represents anxieties present in the humanities today about the future of publishing. The peer review of scholarly books that is at the core of Kriterium, can be seen as a means to an end — to validate and protect the status of scholarly monographs (and edited volumes) — rather than an objective in its own right, even if the value of quality control certainly was part of early negotiations. In retrospective interviews, many participants in the pilot phase voiced their conviction that “the humanistic tradition to write monographs needs to be valued higher” (Hammar 2017, 8). Interestingly, such discussions resonate with Ochsner et al. (2013) identification of types of research in the humanities where “modern” research associated with career-orientation, predictability, interdisciplinarity, and internationalization is positioned against more traditional scholarship that values disciplinarity, autonomy, and local contexts. Kriterium could be seen as an intervention that mediates between these demands through a compromise which embraces increasing expectations of blind and formalized peer review and at the same time protects a largely nationally oriented publishing culture where books play a central role.

## Data Availability

The original contributions presented in the study are included in the article/Supplementary Material, further inquiries can be directed to the corresponding author.

## Footnotes

1
https://www.doabooks.org/en/article/operas-certification-service (https://perma.cc/Z23C-8LQX), (accessed 2021-09-23).

2 While it can be pointed out that internationally, conference proceedings today frequently adopt peer review formats similar to that of journals, this practice is not formalized in the Swedish humanities. Of the edited volumes under scrutiny in this study, several are clearly the result of conferences without necessarily being easily identifiable as being conference proceedings.

3
https://kriterium.se/site/en-about/ (https://perma.cc/4XTP-YFTR), (accessed 2021-09-21).

4 Presently, there is no cost for the individual authors or the publishing houses for submitting manuscripts, although there is a small remuneration for academic coordinators and reviewers covered by the Kriterium consortium.

5 More information can be found on Kriterium’s website: https://www.kriterium.se/site/en-publish/ (https://perma.cc/48EE-PK7D); https://www.kriterium.se/site/en-reviewerguidelines/ (https://perma.cc/6JCS-48D7), (accessed 2021-09-21).

6 In one case (Edv K) the author chose to retract the first submission, but submitted “a more processed contribution,” according to the academic coordinator.

7 If the series editor and the authors/volume editors are in the same department, particular care is made to find reviewers who have no connection to the book’s authors/editors. For guidelines see: https://kriterium.se/site/en-reviewerguidelines/ (https://perma.cc/6JCS-48D7), (accessed 2021-09-21).

8 The publisher can still make an independent decision to publish a book without the Kriterium label.

9
https://kriterium.se/site/en-publish/ (https://perma.cc/48EE-PK7D), (accessed 2021-09-21).
